# Efficacy and safety of rivaroxaban in neonatal catheter-related thrombosis: a single-center retrospective study of 122 cases

**DOI:** 10.7717/peerj.20375

**Published:** 2025-11-20

**Authors:** Rong Zhang, Gang Chen, Wen Hong Cai, Bin Yang, Yun Feng Lin, Teng Hui Zhan

**Affiliations:** 1 Fujian Maternity and Child Health Hospital, College of Clinical Medicine for Obstetrics & Gynecology and Pediatrics, Fujian Medical University, Fuzhou, Fujian, China; 2 Zhongshan Hospital (Xiamen), Fudan University, Xiamen, China; 3 Fujian Key Laboratory of Women and Children’s Critical Diseases Research (Fujian Maternity and Child Health Hospital), Fuzhou, Fujian, China; 4 Fujian Children’s Hospital (Fujian Branch of Shanghai Children’s Medical Center), College of Clinical Medicine for Obstetrics & Gynecology and Pediatrics, Fujian Medical University, Fuzhou, Fujian, China; 5 Clinical Research Center for Pediatric Neurodevelopmental Disease of Fujian Province, Fuzhou, Fujian, China

**Keywords:** Neonate, Catheter-related thrombosis, Rivaroxaban, Anticoagulation therapy, Risk factors

## Abstract

**Background:**

To systematically evaluate the clinical efficacy of rivaroxaban in treating neonatal catheter-related thrombosis (CRT) and analyze risk factors affecting treatment outcomes.

**Methods:**

Clinical data of 122 neonatal CRT patients treated with rivaroxaban from March 2022 to October 2024 at Fujian Provincial Maternal and Child Health Hospital were retrospectively analyzed. The primary outcome was the complete thrombus resolution rate. Multivariate logistic regression was used to analyze risk factors affecting treatment efficacy.

**Results:**

Among 122 patients, the complete thrombus resolution rate was 71.31% (87/122) after 6 weeks of anticoagulation, which significantly increased to 88.52% (108/122) after extending to 3 months (*p* < 0.01). Multivariate logistic regression analysis showed that chemotherapy (OR = 5.48, 95% CI [1.04–28.73], *P* < 0.05) and difficult catheter placement (OR = 12.53, 95% CI [3.13–50.22], *P* < 0.05) were independent risk factors reducing the likelihood of complete thrombus resolution at 3 months. No anticoagulation-related bleeding or other complications were observed during the study period, though the sample size and follow-up period may limit the detection of rare events.

**Conclusion:**

Retrospective data suggest that rivaroxaban is safe and effective in treating neonatal catheter-related thrombosis, with a higher complete thrombus resolution rate observed at 3 months compared to 6 weeks of anticoagulation therapy. Chemotherapy and difficult catheter placement were identified as independent risk factors affecting treatment efficacy. These findings, derived from a single-center retrospective study, require validation through multi-center, prospective, randomized controlled trials.

## Introduction

Venous thromboembolism (VTE) is relatively rare in healthy children; however, its incidence has increased significantly in recent years among children with underlying diseases. Early studies reported an incidence of 37–60 cases per 10,000 hospitalized patients (Raffini et al., 2009), while a recent meta-analysis indicated that hospital-acquired VTE (HA-VTE) accounts for 4.1% of pediatric hospital admissions (Zhou et al., 2024). Unlike adult VTE, where incidence increases with age, pediatric VTE demonstrates a bimodal age distribution, with the first peak occurring during the neonatal period and the second during adolescence (Raffini et al., 2009). Multiple studies have shown that neonates have the highest risk of thrombosis among children (Andrew et al., 1994; Van Ommen et al., 2001). The reported incidence of neonatal venous or arterial thrombosis varies considerably, ranging from 2.4 cases per 1,000 Neonatal Intensive Care Unit(NICU) admissions in the 1990s Canadian registry to 38 cases per 1,000 NICU admissions in recent registries (Van Ommen et al., 2023; Schmidt & Andrew, 1995; El-Naggar et al., 2020). Neonatal CRT is associated with significant morbidity, including risks of pulmonary embolism and post-thrombotic syndrome, underscoring the need for effective and safe anticoagulation therapies.

Central venous catheter (CVC), with their unique advantages, have become crucial lifelines for pediatric patients requiring chemotherapy, blood products, and parenteral nutrition (Ares & Hunter, 2017). However, with the widespread use of CVC, numerous studies have identified them as an independent risk factor for pediatric VTE (Jaffray et al., 2020; Xiong et al., 2025; Mahajerin et al., 2015). Research indicates that over 90% of neonatal venous thrombotic events are catheter-related (Amankwah et al., 2014). The risk of neonatal CRT is further increased by additional factors, including prematurity, sepsis, and congenital heart disease, which calls for targeted therapeutic approaches.

Although controversy exists regarding whether and how to provide anticoagulation for neonatal catheter-related thrombosis (CRT) (Van Ommen et al., 2023; Jones et al., 2019), both the 2012 American College of Chest Physicians Evidence-Based Clinical Practice Guidelines (ACCP9) and the 2018 American Society of Hematology (ASH) guidelines for pediatric venous thromboembolism management recommended heparin-based therapy (including unfractionated and low-molecular-weight heparin) for neonatal CRT (Monagle et al., 2012; Monagle et al., 2018). However, heparin-based anticoagulation has limitations, including the need for frequent monitoring and subcutaneous injections, which can cause pain and bruising (Mohammady, Radmehr & Janani, 2021), challenging patient compliance. While specific data on pediatric compliance with subcutaneous anticoagulation is lacking, even adult compliance rates are suboptimal (McGinnis et al., 2022). With the completion of the EINSTEIN-Jr trial (Male et al., 2020), rivaroxaban has been proven safe and effective for treating pediatric VTE, marking a new era in pediatric VTE. In 2025, the American Society of Hematology/International Society on Thrombosis and Haemostasis updated the guidelines for the treatment of venous thromboembolism in pediatric patients, recommending rivaroxaban as the preferred option for children with VTE (Monagle et al., 2025). Pediatric VTE patients will have access to painless and convenient anticoagulation therapy. The US Food and Drug Administration (FDA) approved rivaroxaban in December 2021 for pediatric VTE, including in neonates, following the EINSTEIN-Jr trial; however, there is still a lack of real-world evidence specifically related to neonatal CRT.

However, it is noteworthy that while the EINSTEIN-Jr trial included 37 (29%) CRT patients under two years of age and conducted a CRT subgroup analysis (Thom et al., 2020), the authors did not specify the detailed age distribution or whether neonates (age < 28 days) were included. Therefore, additional evidence is needed to support the use of rivaroxaban in treating neonatal CRT. This retrospective study investigates the real-world clinical outcomes of rivaroxaban treatment in neonatal CRT and analyzes potential risk factors affecting treatment efficacy, thereby providing evidence to guide clinical practice.

## Materials & Methods

### Ethical approval

This study was approved by the Ethics Committee of Fujian Provincial Maternal and Child Health Hospital (IRB: No. 2024KY301) and conformed to the Declaration of Helsinki. The requirement for informed consent was waived by the Ethics Committee because this retrospective analysis was limited to preexisting data collected as part of the standard care or treatment. Furthermore, data anonymization and privacy were protected.

### Study population

This single-center retrospective study included 122 neonates with CRT out of 14,061 total neonates admitted to Fujian Provincial Maternal and Child Health Hospital between March 2022 and October 2024. The inclusion criteria were: (1) Neonatal period (within 28 days after birth); (2) Clear history of infusion catheter use; (3) Ultrasound-confirmed CRT; (4) Standardized rivaroxaban anticoagulation therapy and follow-up under specialist physician guidance after CRT diagnosis. Exclusion criteria included: (1) Patients with severe diseases affecting coagulation function and metabolism, including end-stage renal disease, liver failure (Child-Pugh C), severe heart failure (NYHA IV), refractory infections, and autoimmune diseases requiring long-term immunosuppressive therapy; (2) Incomplete clinical data; (3) Participation in other clinical trials; (4) Parents/guardians not consenting to follow-up.

### Anticoagulation therapy

Following CRT diagnosis, rivaroxaban was administered at weight-adjusted doses for 6 weeks to 3 months. The rivaroxaban dosing regimen was adjusted to an equivalent 20 mg dose based on body weight: once daily (weight ≥ 30 kg), twice daily (weight 12–30 kg), or three times daily (weight < 12 kg), as specified in the EINSTEIN-Jr trial (Male et al., 2020). Ultrasound examination was performed by trained radiologists using a standardized high-frequency linear probe, with assessments at baseline and 6 weeks to evaluate thrombus size and blood flow. Treatment was discontinued if thrombus resolved completely or extended to 3 months if partial resolution, no resolution, or progression was observed. Catheters were removed when no longer needed, infected, or non-functional.

### Data collection

Researchers collected demographic characteristics of the included patients (age, sex, weight), whether they were born prematurely, primary disease, length of hospital stay, type of medical insurance, whether surgery was performed, type of surgery, whether chemotherapy was administered, central catheter insertion site, catheter type, whether catheter placement was difficult, the location of CRT occurrence, anticoagulation duration, and other relevant information. Additionally, complications such as gastrointestinal bleeding (skin, gastrointestinal tract, oral cavity, intracranial, etc.) and allergic reactions during anticoagulation therapy were also recorded. The primary endpoint of this study was the complete resolution rate of thrombosis. Complete resolution was defined as the previously identified thrombus becoming undetectable and blood flow normalizing. Partial resolution was defined as a reduction in the size of the previously identified thrombus but with persistence. No change in thrombus size or continued absence of blood flow was defined as no resolution. An increase in thrombus size was classified as progression. Difficult catheter placement was defined as two or more attempts at the same site. The study data were sourced from the hospital’s electronic medical record system and laboratory information system, and independently extracted by two trained researchers. Any discrepancies were resolved by a senior physician with advanced qualifications.

### Statistical analysis

Continuous variables are expressed as mean ± standard deviation (for normal distributions) or median (minimum, maximum), while categorical variables are presented as frequencies or percentages. Intergroup comparisons were conducted using the *χ*2 test (for categorical variables), Student’s *t*-test (for continuous variables with a normal distribution), or the Mann–Whitney *U* test (for skewed distributions). Multivariable logistic regression analysis was employed to identify key factors influencing the effectiveness of anticoagulant therapy. A *p*-value <0.05 was considered statistically significant. Statistical analyses were performed using GraphPad Prism 9.5.1 and SPSS 21.0 software (IBM Corp., Armonk, NY, USA).

## Results

### The selection of patients

**Figure 1:**
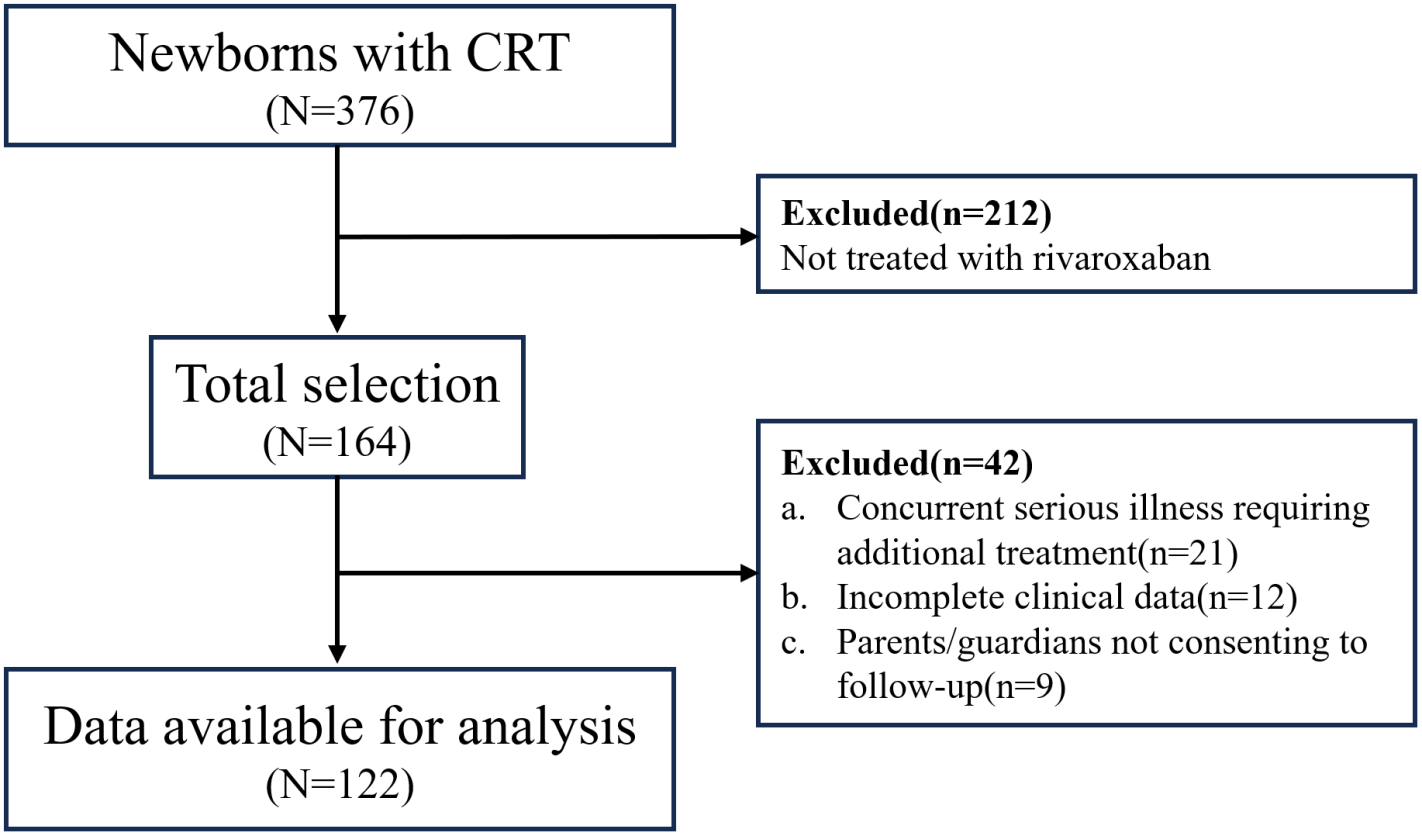
The flowchart of patients’ selection. CRT, catheter-related thrombosis.

During the study period, a total of 376 neonates developed catheter-related thrombosis out of 14,061 neonates admitted to the NICU. After screening according to inclusion and exclusion criteria, 212 patients were excluded due to not receiving rivaroxaban anticoagulation therapy. Among the remaining 164 patients, 21 were excluded due to concurrent serious illnesses, 12 were excluded due to incomplete clinical data, and nine were excluded due to parents/guardians not consenting to follow-up. Finally, 122 patients were included in the data analysis (Fig. 1).

### Baseline characteristics of participants

The study included 122 pediatric patients, predominantly male (63.93%, *n* = 78), with a mean age of 8.25 ± 7.13 days and mean weight of 2854.05 ± 460.42 grams. Premature infants constituted a minority of the cohort (12.30%, *n* = 15). Primary diagnoses showed diverse distribution, with gastrointestinal disorders being most prevalent (32.79%, *n* = 40), followed by respiratory system diseases (22.95%, *n* = 28) and circulatory system disorders (19.67%, *n* = 24). Less common conditions included hematologic disorders (11.48%, *n* = 14), solid tumors (8.20%, *n* = 10), and neurological diseases (4.92%, *n* = 6).

Regarding medical interventions, 69 patients (56.56%) underwent surgical procedures, including gastrointestinal surgery in 38 patients (55.07%), cardiovascular surgery in 18 patients (26.08%), tumor resection in 10 patients (14.50%), respiratory system surgery in 2 patients (2.90%), and neurosurgical procedures in one patient (1.45%). All 10 patients who underwent tumor resection received postoperative chemotherapy (8.20%). The mean hospital length of stay for all included patients was 35.35 ± 30.48 days, with a mean follow-up duration of 102.03 ± 32.66 days.

Healthcare coverage was predominantly through rural medical insurance (54.10%, *n* = 66), with the remaining patients covered by local (22.95%, *n* = 28) and non-local (21.31%, *n* = 26) resident medical insurance programs (Table 1).

**Table 1:** Baseline characteristics of participants.

Characteristics	N
**Total**	122
**Age** (days, mean ± SD)	8.25 ± 7.13
**Gender** (n, %)	
Male	78 (63.93)
Female	44 (36.07)
**Weight** (g, mean ± SD)	2854.05 ± 460.42
**Premature** (n, %)	
Yes	15 (12.30)
No	107 (87.70)
**Type of primary disease** (n, %)	
Hematological disease	14 (11.48)
Digestive system	40 (32.79)
Circulatory system	24 (19.67)
Respiratory system	28 (22.95)
Nervous system	6 (4.92)
Solid tumor	10(8.20)
**Length of hospital stay** (days, mean ± SD)	35.35 ± 30.48
**Type of medical insurance** (n, %)	
Rural medical insurance	66 (54.10)
Local resident medical insurance	28 (22.95)
Non-local resident medical insurance	26 (21.31)
Fully self-funded	2 (1.64)
**Surgery** (n, %)	
Yes	69 (56.56)
No	53 (43.44)
**Type of surgery** (n, %)	
Digestive system	38(55.07)
Circulatory system	18 (26.08)
Respiratory system	2 (2.90)
Nervous system	1 (1.45)
Tumor resection	10 (14.50)
**Chemotherapy** (n, %)	
Yes	10 (8.20)
No	112 (91.80)
**Follow-up time** (days, mean ± SD)	102.03 ± 32.66

### Characteristics of CRT occurrence

Data analysis revealed that the internal jugular vein was the most common site of CRT (45.08%, 55/122), followed by upper extremity veins (36.89%, 45/122), with lower extremity veins showing the lowest incidence (18.03%, 22/122). Regarding catheter types, CRT occurred most frequently with central venous catheters (CVC, 45.90%, 56/122) and peripherally inserted central catheters (PICC, 44.26%, 54/122), while implanted ports (PORT) were less common (9.84%, 12/122). Notably, difficult catheter placement was documented in 27.87% (34/122) of cases (Table 2).

**Table 2:** Characteristics of catheter-related thrombosis.

Characteristics	N
**Catheter type** (n, %)	
PICC	54 (44.26)
PORT	12 (9.84)
CVC	56 (45.90)
**Difficulties in catheter placement** (n, %)	
Yes	34 (27.87)
No	88 (72.13)
**Site of CRT** (n, %)	
Upper limb veins	45 (36.89)
Internal jugular vein	55 (45.08)
Lower limb veins	22 (18.03)

**Notes.**

PICC, Peripherally Inserted Central Venous Catheterization; PORT, Implantable Venous Access Port System; CVC, Central Venous Catheterization; CRT, Catheter-Related Thrombosis.

### Anticoagulation treatment outcomes and complications

**Figure 2:**
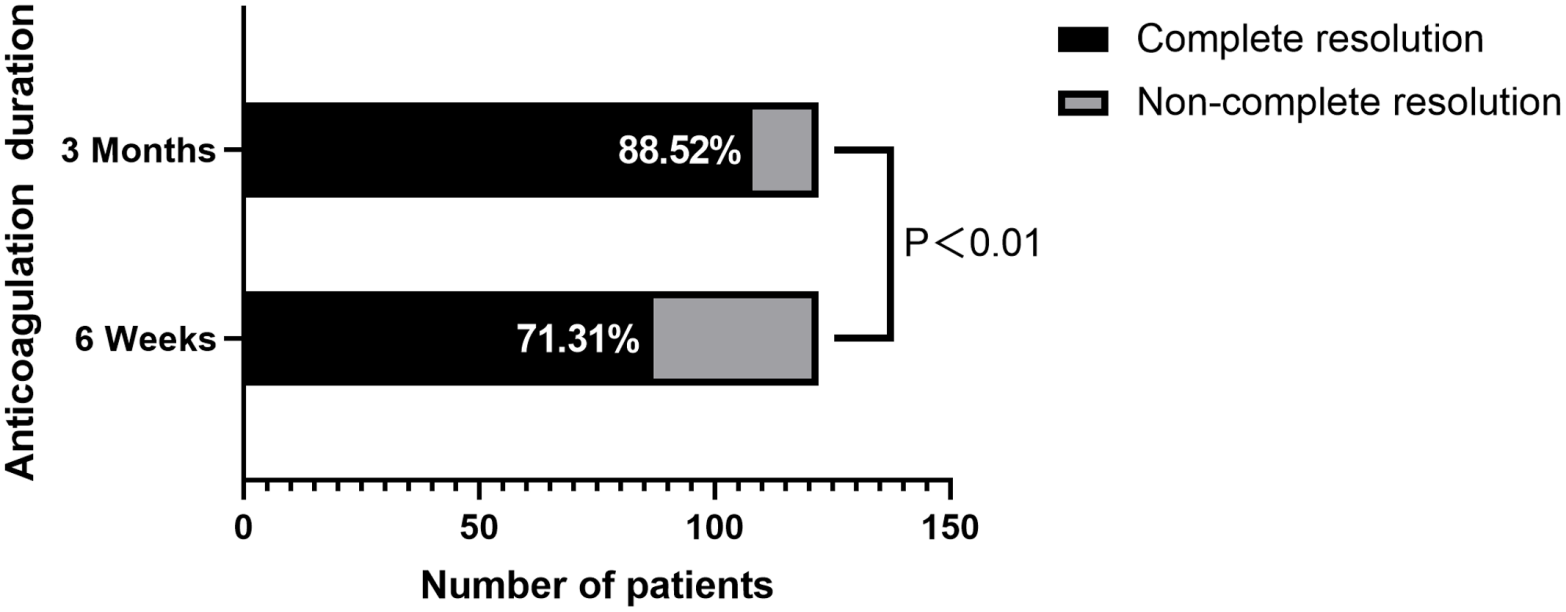
Comparison of complete resolution rates.

Among the 122 neonates with catheter-related thrombosis who received anticoagulation therapy, the majority (71.31%, 87/122) underwent 6 weeks of treatment, while the remaining patients (28.69%, 35/122) extended to 3 months. After 6 weeks of anticoagulation, complete thrombus resolution was achieved in 71.31% (87/122) of patients, partial resolution in 27.05% (33/122), and thrombus progression in only 1.64% (2/122). After extending anticoagulation to 3 months (including patients from 6 weeks of anticoagulation), the complete thrombus resolution rate significantly improved to 88.52% (108/122), which was statistically significant compared to the 6-week anticoagulation thrombus resolution rate of 71.31% (*p* < 0.01, Fig. 2). The partial thrombus resolution rate decreased to 11.48% (14/122). Subgroup analysis by catheter type revealed significant variations in thrombus resolution: CVC (*n* = 56) showed the highest complete thrombus resolution rate of 92.86% (52/56), followed by PICC (*n* = 54) with a complete thrombus resolution rate of 88.89% (48/54), while PORT (*n* = 12) demonstrated a notably lower complete thrombus resolution rate of 66.67% (8/12). Statistical analysis using chi-square test showed no significant difference between CVC and PICC (*χ*^2^ = 0.536, *p* = 0.464), indicating similar performance, but PORT exhibited significantly different resolution patterns (CVC *vs* PORT: *χ*^2^ = 8.243, *p* = 0.004; PICC *vs* PORT: *χ*^2^ = 6.728, *p* = 0.010). Partial thrombus reduction rates varied, the PORT group showed 33.33% (4/12), the PICC group 11.11% (6/54), and the CVC group 7.14% (4/56). This highlights the potential differences in thrombus response among different catheter types. No thrombus progression or anticoagulation-related adverse events (such as allergic reactions, bleeding, or recurrence) were observed during follow-up (Table 3).

**Table 3:** Effects of anticoagulant therapy and complications

Characteristics	N
**Anticoagulation time** (n, %)	
6 weeks	87 (71.31)
3 months	35 (28.69)
**Six weeks after anticoagulation** (n, %)	
Complete resolution	87 (71.31)
Partial resolution	33 (27.05)
No resolution	0
Progression	2 (1.64)
**Three months after anticoagulation** (n, %)	
Complete resolution	108 (88.52)
Partial resolution	14 (11.48)
No resolution	0
Progression	0
**Resolution rates by catheter type** (n, %)	
**PICC**	
Complete resolution	48 (88.89)
Partial resolution	6 (11.11)
**PORT**	
Complete resolution	8 (66.67)
Partial resolution	4 (33.33)
**CVC**	
Complete resolution	52 (92.86)
Partial resolution	4 (7.14)
**Complications** (n, %)	
Allergy	0
Bleeding associated with anticoagulant therapy	0
Recurrence	0

### Regression analysis of risk factors affecting anticoagulation treatment efficacy

For the 6-week anticoagulation treatment, a multivariate logistic regression analysis included factors such as age, gender, weight, premature infant status, type of primary disease, length of hospital stays, type of medical insurance, surgery, type of surgery, chemotherapy, central venous catheter position, Catheter type, difficulty in catheter placement, and site of CRT. The results showed that chemotherapy is an independent risk factor influencing the effectiveness of anticoagulation treatment for neonatal catheter-related thrombosis. Specifically, patients receiving chemotherapy demonstrated a 6.05-fold higher risk of persistent thrombosis compared to those without chemotherapy (OR = 6.05, 95% CI [1.32–28.49], *P* = 0.02). Although surgery type (OR = 0.51, 95% CI [0.12–2.20], *P* = 0.37), catheter type (OR = 1.50, 95% CI [0.79–2.84], *P* = 0.22), and difficult catheter placement (OR = 2.95, 95% CI [0.86–10.03], *P* = 0.08) showed potential associations in univariate analysis, they did not reach statistical significance in multivariate analysis. Factors such as age, gender, weight, premature infant status, type of primary disease, length of hospital stays, type of medical insurance, position of the central venous catheter, and site of CRT did not show a significant impact on the effectiveness of anticoagulant therapy (Table 4).

**Table 4:** Risk factors affecting anticoagulation treatment efficacy (6 weeks).

Variables	Univariate analysis		Multivariate analysis		
	t/*χ*2	*P*-value	OR	95% CI	*P*-value
Age	−0.72	0.47	NA	NA	NA
Gender	0.98	0.32	NA	NA	NA
Weight	−1.56	0.12	NA	NA	NA
Premature	0.63	0.13	NA	NA	NA
Type of primary disease	10.20	0.07	NA	NA	NA
Length of hospital stay	0.78	0.44	NA	NA	NA
Type of medical insurance	3.53	0.32	NA	NA	NA
Surgery	0.24	0.63	NA	NA	NA
Type of surgery	9.60	**<0**.**05**	0.51	0.12, 2.20	0.37
Chemotherapy	9.01	**<0**.**05**	6.05	1.32, 28.49	**0**.**02**
Central venous catheter position	1.69	0.43	NA	NA	NA
Catheter type	9.93	**<0**.**05**	1.50	0.79, 2.84	0.22
Difficulties in catheter placement	7.78	**<0**.**05**	2.95	0.86, 10.03	0.08
Site of CRT	1.69	0.43	NA	NA	NA

**Notes.**

OR, Odds ratio; NA, Not Applicable; CI, Confidence Interval; CRT, Catheter-Related Thrombosis. Bold values indicate statistical significance.

For the 3-month anticoagulation treatment, a multivariate logistic regression analysis included factors such as age, gender, weight, premature infant status, type of primary disease, length of hospital stays, type of medical insurance, surgery, type of surgery, chemotherapy, central venous catheter position, Catheter type, difficulty in catheter placement, and site of CRT. The results showed that chemotherapy (OR 5.48, 95% CI [1.04–28.73], *p* < 0.05) and catheter insertion difficulty (OR 12.53, 95% CI [3.13–50.22], *p* < 0.05) were independent significant factors affecting the effectiveness of anticoagulation treatment. Although catheter type (OR = 0.68, 95% CI [0.33–1.41], *P* = 0.30) showed a potential association in univariate analysis, it did not achieve statistical significance in multivariate analysis. Factors such as age, gender, weight, premature infant status, type of primary disease, length of hospital stays, type of medical insurance, surgery, type of surgery, central venous catheter position, and site of CRT did not show a significant impact on the effectiveness of anticoagulation treatment (Table 5).

**Table 5:** Risk factors affecting anticoagulation treatment efficacy (3 months).

Variables	Univariate analysis		Multivariate analysis		
	t/*χ*2	*P*-value	OR	95% CI	*P*-value
Age	−1.08	0.29	NA	NA	NA
Gender	0.39	0.54	NA	NA	NA
Weight	−0.55	0.59	NA	NA	NA
Premature	0.39	0.53	NA	NA	NA
Type of primary disease	9.71	0.08	NA	NA	NA
Length of hospital stay	1.20	0.25	NA	NA	NA
Type of medical insurance	3.64	0.45	NA	NA	NA
Surgery	0.38	0.54	NA	NA	NA
Type of surgery	7.80	0.10	NA	NA	NA
Chemotherapy	8.73	**<0**.**05**	5.48	1.04, 28.73	**<0**.**05**
Central venous catheter position	3.67	0.16	NA	NA	NA
Catheter type	6.69	**<0**.**05**	0.68	0.33, 1.41	0.30
Difficulties in catheter placement	20.23	**<0**.**05**	12.53	3.13, 50.22	**<0**.**05**
Site of CRT	3.67	0.16	NA	NA	NA

**Notes.**

OR, Odds ratio; NA, Not Applicable; CI, Confidence Interval; CRT, Catheter-Related Thrombosis. Bold values indicate statistical significance.

## Discussion

This study represents the first systematic evaluation of rivaroxaban treatment for neonatal CRT in a single-center retrospective setting, including 122 neonates with a mean follow-up duration of 102.03 ± 32.66 days. Using rivaroxaban anticoagulation as the primary intervention, with complete thrombus resolution rate as the main outcome measure, the study demonstrated that complete resolution was achieved in 71.31% (87/122) of patients after 6 weeks of anticoagulation, significantly improving to 88.52% (108/122) when extended to 3 months (*p* < 0.01). Multivariate logistic regression analysis identified chemotherapy (OR = 5.48, 95% CI [1.04–28.73]) and difficult catheter placement (OR = 12.53, 95% CI [3.13–50.22]) as independent risk factors affecting treatment efficacy.

The true incidence of neonatal catheter-related thrombosis remains uncertain. Recently, a prospective, multicenter, observational study based on the Dutch national guidelines for managing neonatal catheter-related venous thrombosis reported that 115 (0.4%) of 29,074 neonates admitted to 10 NICUs between 2014 and 2019 developed CRT (Van Ommen et al., 2023). This incidence remained stable throughout the six-year study period, consistent with previous reports (Schmidt & Andrew, 1995; Chojnacka et al., 2022). However, this figure likely underestimates the true prevalence. Firstly, selection bias makes it challenging to include all CRT cases in registries (Van Ommen et al., 2023). More importantly, current guidelines universally recommend against routine ultrasound screening for venous thrombosis in asymptomatic patients with central venous catheters (Monagle et al., 2012; Monagle et al., 2018), a principle strictly adhered to at our center. While we acknowledge the lack of large-scale randomized controlled trials supporting the clinical value of routine screening and recognize that such screening might lead to unnecessary medical resource utilization and patient anxiety, this approach inevitably results in underestimation of neonatal CRT incidence.

The optimal management of neonatal CRT remains unclear. There is general consensus that most studies do not support pharmacological prophylaxis for pediatric CRT, nor demonstrate that anticoagulants can reduce the risk of catheter-related thrombosis in children (Pelland-Marcotte et al., 2020; Vidal et al., 2014). Regarding the natural course of established pediatric CRT, studies have observed spontaneous resolution. A prospective cohort study showed that among 128 neonates with catheter-related internal jugular vein thrombosis, 67% experienced spontaneous resolution within one month after discharge without anticoagulation, increasing to 73% at 6 months (Xiong et al., 2025). Another prospective study following 24 of 32 children with CRT for two years without anticoagulation found residual thrombosis in only three patients at follow-up (Jones et al., 2019).

However, several studies have indicated that CRT can lead to severe, potentially life-threatening complications. Limited data exist regarding neonatal VTE mortality rates, which reportedly range from 0.9% to 3.7% (Van Ommen et al., 2023). Cases of pulmonary embolism following pediatric CRT have been documented (Jaffray et al., 2022). A systematic review revealed that approximately 3% of infants under three months experienced recurrent VTE, while a retrospective study of catheter-related upper extremity VTE in children demonstrated that 16% of neonates developed mild post-thrombotic syndrome, manifesting as limb swelling and collateral vein formation (Van Ommen et al., 2023; Ting et al., 2021). Post-CRT pulmonary embolism poses a life-threatening risk, while post-thrombotic syndrome can significantly impact patients’ quality of life. Consequently, we advocate for aggressive anticoagulation therapy in neonatal CRT patients to minimize or prevent these complications from affecting growth and quality of life.

In our study, complete thrombus resolution was achieved in 71.31% of patients after 6 weeks of anticoagulation therapy and in 88.52% after 3 months. Although direct comparisons are not feasible, these resolution rates substantially exceed the spontaneous resolution rates reported in existing literature. Currently, there is insufficient evidence to determine whether 3-month anticoagulation therapy offers superior clinical benefits compared to a 6-week regimen. While our findings appear to favor the 3-month duration, we advocate for careful consideration in selecting the anticoagulation period. Based on available evidence, we recommend initiating a 6-week anticoagulation course, discontinuing treatment if complete thrombus resolution is achieved while maintaining close follow-up, and extending therapy to 3 months if resolution is incomplete or deterioration occurs, thereby minimizing residual thrombosis. Given its high resolution rates and low bleeding risk, rivaroxaban may represent a viable alternative to low-molecular-weight heparin (LMWH), potentially warranting consideration in future neonatal CRT treatment guidelines, pending prospective validation. The absence of recurrent thrombosis in our study population may be attributed to our aggressive anticoagulation approach.

Another widely discussed concern is the complications associated with anticoagulation therapy, with bleeding being the most prominent and common. A recent literature review on LMWH treatment for neonatal thrombosis revealed various manifestations of bleeding episodes, including occult blood in stool, injection site bleeding, intracranial hemorrhage, and epistaxis. Reported bleeding rates varied from 0% to 30%, with differences attributed to varying definitions of bleeding, sample sizes, and study populations. Subsequent stratified analysis indicated a bleeding rate of approximately 3.9% in neonates (Ting et al., 2021). In the NEOCLOT study, 10 out of 63 infants receiving LMWH treatment developed bleeding complications (15.8%). In contrast, the EINSTEIN-Jr study (Male et al., 2020) and EINSTEIN-Jr CVC-VTE study (Thom et al., 2020) reported bleeding rates of 3% and 2.4%, respectively, for rivaroxaban in treating pediatric venous thromboembolism and catheter-related venous thrombosis, substantially lower than the bleeding rates associated with LMWH. In our study, no anticoagulation-related bleeding events were observed, although this finding may be attributed to our relatively small sample size and short follow-up period.

Endothelial injury, local turbulent flow around venous catheters, reduced blood flow velocity, and alterations in blood composition are the primary factors contributing to CRT (Gray et al., 2012). Previous systematic analyses have identified several risk factors for CRT, including elevated D-dimer levels, catheter insertion site, catheter type, number of lumens, catheter dwell time, central line-associated bloodstream infections, history of thrombosis, gastrointestinal/hepatic diseases, hematologic disorders, malignancy, parenteral nutrition, hemodialysis, extracorporeal membrane oxygenation, and cardiac catheterization (Fu et al., 2024; Tian et al., 2021). Additionally, a prospective observational study of pediatric surgical patients with central venous catheters demonstrated that more than two insertion attempts increased the risk of thrombosis (Kim et al., 2022). Currently, no studies have identified factors influencing the effectiveness of anticoagulation therapy in pediatric CRT.

Our findings suggest that chemotherapy (OR = 6.05, 95% CI [1.32–28.49], *P* = 0.02) may be an independent risk factor affecting treatment outcomes at 6 weeks, while both chemotherapy (OR = 5.48, 95% CI [1.04–28.73], *P* < 0.05) and difficult catheter placement (OR = 12.53, 95% CI [3.13–50.22], *P* < 0.05) emerged as independent risk factors affecting treatment outcomes at 3 months of anticoagulation therapy. We propose the following potential mechanisms: First, patients receiving chemotherapy were exclusively malignancy cases, where overexpression of certain cytokines by tumor cells can induce platelet aggregation or vascular endothelial inflammatory responses, thereby promoting blood coagulation (Christison-Lagay et al., 2024). This represents one of the primary causes of high VTE incidence in cancer patients and may also influence anticoagulation treatment efficacy. Second, multiple vessel punctures necessitated by difficult catheter placement inevitably increase vascular wall damage. Given the delicate nature of neonatal vessels, repeated punctures can affect blood flow in small vessels (Kim et al., 2022), thereby increasing CRT occurrence and compromising anticoagulation treatment effectiveness. Literature suggests that ultrasound-guided central venous catheter placement can reduce the number of puncture attempts (Oulego-Erroz et al., 2018), providing an effective solution to this challenge.

Several limitations exist in this study. First, as a single-center retrospective study, the generalizability and external validity of our findings require validation through multi-center data. Second, since the study population primarily consisted of Chinese neonates, caution should be exercised when extrapolating these results to other ethnic populations. Third, as an observational study, we could only demonstrate associations between rivaroxaban treatment and thrombus resolution, rather than establish causal relationships. Furthermore, although we adjusted for known confounding factors, there may be unidentified confounders influencing our results. Finally, the inability to conduct long-term follow-up for all patients limited our assessment of the treatment’s long-term outcomes. This limitation underscores the need for prospective studies with extended follow-up to evaluate long-term outcomes, such as post-thrombotic syndrome and recurrent VTE, which significantly impact neonatal health.

## Conclusions

Retrospective data from a single-center study suggest that rivaroxaban is potentially safe and effective in treating neonatal catheter-related thrombosis, though the small sample size and short follow-up may limit detection of rare complications, with a higher complete thrombus resolution rate observed at 3 months compared to 6 weeks of anticoagulation therapy. Chemotherapy and difficult catheter placement were identified as independent risk factors affecting treatment efficacy. These findings position rivaroxaban as a potential alternative to low-molecular-weight heparin, warranting consideration in future neonatal CRT treatment guidelines. Further studies are needed to assess long-term outcomes, such as post-thrombotic syndrome and recurrent VTE, through multi-center, prospective, randomized controlled trials.

## Supplemental Information

10.7717/peerj.20375/supp-1 Supplemental Information 1 Raw data

10.7717/peerj.20375/supp-2 Supplemental Information 2 STROBE checklist

## Additional Information and Declarations

### Data Availability

The following information was supplied regarding data availability: The raw measurements are available in the Supplementary File.
