# Supplementary material for: Efficacy and safety of rivaroxaban in neonatal catheter-related thrombosis: a single-center retrospective study of 122 cases
Source: PeerJ. 2025 Nov 20;13:e20375. doi: 10.7717/peerj.20375 (PMC12640641; doi:10.7717/peerj.20375)
Supplement: Supplemental Information 2 [file peerj-13-20375-s002.docx]

STROBE Statement—checklist of items that should be included in reports of observational studies

|  | Item No. | Recommendation | Page  No. | Relevant text from manuscript |
| --- | --- | --- | --- | --- |
| **Title and abstract** | 1 | (*a*) Indicate the study’s design with a commonly used term in the title or the abstract | 1 | A Single-center Retrospective Study |
|  |  | (*b*) Provide in the abstract an informative and balanced summary of what was done and what was found | 2 | Conclusion: Retrospective data suggest that rivaroxaban is safe and effective in treating neonatal catheter-related thrombosis, with a higher complete thrombus resolution rate observed at 3 months compared to 6 weeks of anticoagulation therapy. Chemotherapy and difficult catheter placement were identified as independent risk factors affecting treatment efficacy. However, these findings require validation through multi-center, prospective, randomized controlled trials. |
| Introduction | | | |  |
| Background/rationale | 2 | Explain the scientific background and rationale for the investigation being reported | 2,3 | Although controversy exists regarding whether and how to provide anticoagulation for neonatal catheter-related thrombosis (CRT) [5,13], the 2012 American College of Chest Physicians Evidence-Based Clinical Practice Guidelines (ACCP9) recommended heparin-based therapy (including unfractionated and low-molecular-weight heparin) for neonatal CRT [14]. The 2018 American Society of Hematology (ASH) guidelines for VTE management proposed similar treatment protocols [15]. However, heparin-based anticoagulation has limitations, including the need for frequent monitoring and subcutaneous injections, which can cause pain and bruising [16], challenging patient compliance. While specific data on pediatric compliance with subcutaneous anticoagulation is lacking, even adult compliance rates are suboptimal [17]. With the completion of the EINSTEIN-Jr trial [18], rivaroxaban has been proven safe and effective for treating pediatric VTE, marking a new era in pediatric VTE treatment by offering painless and convenient anticoagulation therapy. |
| Objectives | 3 | State specific objectives, including any prespecified hypotheses | 3 | This retrospective study investigates the real-world clinical outcomes of rivaroxaban treatment in neonatal CRT and analyzes potential risk factors affecting treatment efficacy, thereby providing evidence to guide clinical practice. |
| Methods | | | |  |
| Study design | 4 | Present key elements of study design early in the paper | 3 | 2. Study population  This single-center retrospective study included neonates with CRT admitted to Fujian Provincial Maternal and Child Health Hospital between March 2022 and October 2024. The inclusion criteria were: (1) Neonatal period (within 28 days after birth); (2) Clear history of infusion catheter use; (3) Ultrasound-confirmed CRT; (4) Standardized rivaroxaban anticoagulation therapy and follow-up under specialist physician guidance after CRT diagnosis. Exclusion criteria included: (1) Concurrent serious illness requiring additional treatment; (2) Incomplete clinical data; (3) Participation in other clinical trials; (4) Parents/guardians not consenting to follow-up.  3. Anticoagulation Therapy  Following CRT diagnosis, rivaroxaban was administered at weight-adjusted doses for 6 weeks to 3 months. Ultrasound examination was performed after 6 weeks of anticoagulation; treatment was discontinued if thrombus resolved completely or extended to 3 months if thrombus persisted. Catheters were removed when no longer needed, infected, or non-functional. |
| Setting | 5 | Describe the setting, locations, and relevant dates, including periods of recruitment, exposure, follow-up, and data collection | 3,4 | 2. Study population  This single-center retrospective study included neonates with CRT admitted to Fujian Provincial Maternal and Child Health Hospital between March 2022 and October 2024. The inclusion criteria were: (1) Neonatal period (within 28 days after birth); (2) Clear history of infusion catheter use; (3) Ultrasound-confirmed CRT; (4) Standardized rivaroxaban anticoagulation therapy and follow-up under specialist physician guidance after CRT diagnosis. Exclusion criteria included: (1) Concurrent serious illness requiring additional treatment; (2) Incomplete clinical data; (3) Participation in other clinical trials; (4) Parents/guardians not consenting to follow-up.  3. Anticoagulation Therapy  Following CRT diagnosis, rivaroxaban was administered at weight-adjusted doses for 6 weeks to 3 months. Ultrasound examination was performed after 6 weeks of anticoagulation; treatment was discontinued if thrombus resolved completely or extended to 3 months if thrombus persisted. Catheters were removed when no longer needed, infected, or non-functional.  4. Data Collection  Researchers collected demographic characteristics of the included patients (age, sex, weight), whether they were born prematurely, primary disease, length of hospital stay, type of medical insurance, whether surgery was performed, type of surgery, whether chemotherapy was administered, central catheter insertion site, catheter type, whether catheter placement was difficult, the location of CRT occurrence, anticoagulation duration, and other relevant information. Additionally, complications such as gastrointestinal bleeding (skin, gastrointestinal tract, oral cavity, intracranial, etc.) and allergic reactions during anticoagulation therapy were also recorded. The primary endpoint of this study was the complete resolution rate of thrombosis. Complete resolution was defined as the previously identified thrombus becoming undetectable and blood flow normalizing. Partial resolution was defined as a reduction in the size of the previously identified thrombus but with persistence. No change in thrombus size or continued absence of blood flow was defined as no resolution. An increase in thrombus size was classified as progression. Difficult catheter placement was defined as two or more attempts at the same site. The study data were sourced from the hospital’s electronic medical record system and laboratory information system, and independently extracted by two trained researchers. Any discrepancies were resolved by a senior physician with advanced qualifications. |
| Participants | 6 | (*a*) *Cohort study*—Give the eligibility criteria, and the sources and methods of selection of participants. Describe methods of follow-up  *Case-control study*—Give the eligibility criteria, and the sources and methods of case ascertainment and control selection. Give the rationale for the choice of cases and controls  *Cross-sectional study*—Give the eligibility criteria, and the sources and methods of selection of participants | 3 | The inclusion criteria were: (1) Neonatal period (within 28 days after birth); (2) Clear history of infusion catheter use; (3) Ultrasound-confirmed CRT; (4) Standardized rivaroxaban anticoagulation therapy and follow-up under specialist physician guidance after CRT diagnosis. |
|  |  | (*b*) *Cohort study*—For matched studies, give matching criteria and number of exposed and unexposed  *Case-control study*—For matched studies, give matching criteria and the number of controls per case |  | N/A |
| Variables | 7 | Clearly define all outcomes, exposures, predictors, potential confounders, and effect modifiers. Give diagnostic criteria, if applicable | 3 | Following CRT diagnosis, rivaroxaban was administered at weight-adjusted doses for 6 weeks to 3 months. Ultrasound examination was performed after 6 weeks of anticoagulation; treatment was discontinued if thrombus resolved completely or extended to 3 months if thrombus persisted. Catheters were removed when no longer needed, infected, or non-functional. |
| Data sources/ measurement | 8* | For each variable of interest, give sources of data and details of methods of assessment (measurement). Describe comparability of assessment methods if there is more than one group |  | N/A |
| Bias | 9 | Describe any efforts to address potential sources of bias | 4 | The study data were sourced from the hospital’s electronic medical record system and laboratory information system, and independently extracted by two trained researchers. Any discrepancies were resolved by a senior physician with advanced qualifications. |
| Study size | 10 | Explain how the study size was arrived at | 3 | This single-center retrospective study included neonates with CRT admitted to Fujian Provincial Maternal and Child Health Hospital between March 2022 and October 2024. The inclusion criteria were: (1) Neonatal period (within 28 days after birth); (2) Clear history of infusion catheter use; (3) Ultrasound-confirmed CRT; (4) Standardized rivaroxaban anticoagulation therapy and follow-up under specialist physician guidance after CRT diagnosis. Exclusion criteria included: (1) Concurrent serious illness requiring additional treatment; (2) Incomplete clinical data; (3) Participation in other clinical trials; (4) Parents/guardians not consenting to follow-up. |

Continued on next page

| Quantitative variables | 11 | Explain how quantitative variables were handled in the analyses. If applicable, describe which groupings were chosen and why | 4 | Continuous variables are expressed as mean ± standard deviation (for normal distributions) or median (minimum, maximum), while categorical variables are presented as frequencies or percentages. Intergroup comparisons were conducted using the χ2 test (for categorical variables), Student's t-test (for continuous variables with a normal distribution), or the Mann-Whitney U test (for skewed distributions). Multivariable logistic regression analysis was employed to identify key factors influencing the effectiveness of anticoagulant therapy. A p-value <0.05 was considered statistically significant. Statistical analyses were performed using GraphPad Prism 9.5.1 and SPSS 21.0 software. |
| --- | --- | --- | --- | --- |
| Statistical methods | 12 | (*a*) Describe all statistical methods, including those used to control for confounding | 4 | Continuous variables are expressed as mean ± standard deviation (for normal distributions) or median (minimum, maximum), while categorical variables are presented as frequencies or percentages. Intergroup comparisons were conducted using the χ2 test (for categorical variables), Student's t-test (for continuous variables with a normal distribution), or the Mann-Whitney U test (for skewed distributions). Multivariable logistic regression analysis was employed to identify key factors influencing the effectiveness of anticoagulant therapy. A p-value <0.05 was considered statistically significant. Statistical analyses were performed using GraphPad Prism 9.5.1 and SPSS 21.0 software. |
|  |  | (*b*) Describe any methods used to examine subgroups and interactions | 4 | Continuous variables are expressed as mean ± standard deviation (for normal distributions) or median (minimum, maximum), while categorical variables are presented as frequencies or percentages. Intergroup comparisons were conducted using the χ2 test (for categorical variables), Student's t-test (for continuous variables with a normal distribution), or the Mann-Whitney U test (for skewed distributions). Multivariable logistic regression analysis was employed to identify key factors influencing the effectiveness of anticoagulant therapy. A p-value <0.05 was considered statistically significant. Statistical analyses were performed using GraphPad Prism 9.5.1 and SPSS 21.0 software. |
|  |  | (*c*) Explain how missing data were addressed | 4 | Continuous variables are expressed as mean ± standard deviation (for normal distributions) or median (minimum, maximum), while categorical variables are presented as frequencies or percentages. Intergroup comparisons were conducted using the χ2 test (for categorical variables), Student's t-test (for continuous variables with a normal distribution), or the Mann-Whitney U test (for skewed distributions). Multivariable logistic regression analysis was employed to identify key factors influencing the effectiveness of anticoagulant therapy. A p-value <0.05 was considered statistically significant. Statistical analyses were performed using GraphPad Prism 9.5.1 and SPSS 21.0 software. The study data were sourced from the hospital’s electronic medical record system and laboratory information system, and independently extracted by two trained researchers. Any discrepancies were resolved by a senior physician with advanced qualifications. |
|  |  | (*d*) *Cohort study*—If applicable, explain how loss to follow-up was addressed  *Case-control study*—If applicable, explain how matching of cases and controls was addressed  *Cross-sectional study*—If applicable, describe analytical methods taking account of sampling strategy |  | N/A |
|  |  | (*e*) Describe any sensitivity analyses |  | N/A |
| Results | | | | |
| Participants | 13* | (a) Report numbers of individuals at each stage of study—eg numbers potentially eligible, examined for eligibility, confirmed eligible, included in the study, completing follow-up, and analysed |  | N/A |
|  |  | (b) Give reasons for non-participation at each stage |  | N/A |
|  |  | (c) Consider use of a flow diagram |  | N/A |
| Descriptive data | 14* | (a) Give characteristics of study participants (eg demographic, clinical, social) and information on exposures and potential confounders |  | N/A |
|  |  | (b) Indicate number of participants with missing data for each variable of interest |  | N/A |
|  |  | (c) *Cohort study*—Summarise follow-up time (eg, average and total amount) |  | N/A |
| Outcome data | 15* | *Cohort study*—Report numbers of outcome events or summary measures over time |  | N/A |
|  |  | *Case-control study—*Report numbers in each exposure category, or summary measures of exposure |  | N/A |
|  |  | *Cross-sectional study—*Report numbers of outcome events or summary measures |  | N/A |
| Main results | 16 | (*a*) Give unadjusted estimates and, if applicable, confounder-adjusted estimates and their precision (eg, 95% confidence interval). Make clear which confounders were adjusted for and why they were included | 5，6 | For the 6-week anticoagulation treatment, multivariate logistic regression analysis identified chemotherapy as an independent risk factor influencing the effectiveness of anticoagulation treatment for neonatal catheter-related thrombosis. Specifically, patients receiving chemotherapy demonstrated a 6.05-fold higher risk of CRT compared to those without chemotherapy (OR=6.05, 95%CI: 1.32-28.49, P=0.02). Although surgery type (OR=0.51, 95%CI: 0.12-2.20, P=0.37), catheter type (OR=1.50, 95%CI: 0.79-2.84, P=0.22), and difficult catheter placement (OR=2.95, 95%CI: 0.86-10.03, P=0.08) showed potential associations in univariate analysis, they did not reach statistical significance in multivariate analysis (Table 4).  For the 3-month anticoagulation treatment, multivariate logistic regression analysis revealed both chemotherapy and difficult catheter placement as independent risk factors affecting treatment efficacy. The study found that patients undergoing chemotherapy exhibited a 5.48-fold higher risk of thrombosis compared to those without chemotherapy (OR=5.48, 95%CI: 1.04-28.73, P<0.05). More critically, difficult catheter placement significantly increased the risk of persistent thrombosis, with affected patients showing a 12.53-fold higher risk compared to those with successful placement (OR=12.53, 95%CI: 3.13-50.22, P<0.05). While catheter type (OR=0.68, 95%CI: 0.33-1.41, P=0.30) showed potential association in univariate analysis, it did not achieve statistical significance in multivariate analysis (Table 5). |
|  |  | (*b*) Report category boundaries when continuous variables were categorized |  | N/A |
|  |  | (*c*) If relevant, consider translating estimates of relative risk into absolute risk for a meaningful time period | 5，6 | For the 6-week anticoagulation treatment, multivariate logistic regression analysis identified chemotherapy as an independent risk factor influencing the effectiveness of anticoagulation treatment for neonatal catheter-related thrombosis. Specifically, patients receiving chemotherapy demonstrated a 6.05-fold higher risk of CRT compared to those without chemotherapy (OR=6.05, 95%CI: 1.32-28.49, P=0.02). Although surgery type (OR=0.51, 95%CI: 0.12-2.20, P=0.37), catheter type (OR=1.50, 95%CI: 0.79-2.84, P=0.22), and difficult catheter placement (OR=2.95, 95%CI: 0.86-10.03, P=0.08) showed potential associations in univariate analysis, they did not reach statistical significance in multivariate analysis (Table 4).  For the 3-month anticoagulation treatment, multivariate logistic regression analysis revealed both chemotherapy and difficult catheter placement as independent risk factors affecting treatment efficacy. The study found that patients undergoing chemotherapy exhibited a 5.48-fold higher risk of thrombosis compared to those without chemotherapy (OR=5.48, 95%CI: 1.04-28.73, P<0.05). More critically, difficult catheter placement significantly increased the risk of persistent thrombosis, with affected patients showing a 12.53-fold higher risk compared to those with successful placement (OR=12.53, 95%CI: 3.13-50.22, P<0.05). While catheter type (OR=0.68, 95%CI: 0.33-1.41, P=0.30) showed potential association in univariate analysis, it did not achieve statistical significance in multivariate analysis (Table 5). |

Continued on next page

| Other analyses | 17 | Report other analyses done—eg analyses of subgroups and interactions, and sensitivity analyses | 5 | Among the 122 neonates with catheter-related thrombosis who received anticoagulation therapy, the majority (71.31%, 87/122) underwent 6 weeks of treatment, while the remaining patients (28.69%, 35/122) extended to 3 months. After 6 weeks of anticoagulation, complete thrombus resolution was achieved in 71.31% (87/122) of patients, partial resolution in 27.05% (33/122), and thrombus progression in only 1.64% (2/122). Following extension to 3 months of anticoagulation, the complete resolution rate significantly improved to 88.52% (108/122), showing statistical significance compared to the 6-week resolution rate (71.31%, p<0.01, Figure 2). The partial resolution rate decreased to 11.48% (14/122), with no observed thrombus progression or anticoagulation-related adverse events (such as allergic reactions, bleeding, or recurrence) (Table 3). |
| --- | --- | --- | --- | --- |
| Discussion | | | | |
| Key results | 18 | Summarise key results with reference to study objectives | 6 | This study represents the first systematic evaluation of rivaroxaban treatment for neonatal CRT in a single-center retrospective setting, including 122 neonates with a mean follow-up duration of 102.03 ± 32.66 days. Using rivaroxaban anticoagulation as the primary intervention, with complete thrombus resolution rate as the main outcome measure, the study demonstrated that complete resolution was achieved in 71.31% (87/122) of patients after 6 weeks of anticoagulation, significantly improving to 88.52% (108/122) when extended to 3 months (p<0.01). Multivariate logistic regression analysis identified chemotherapy (OR=5.48, 95%CI: 1.04-28.73) and difficult catheter placement (OR=12.53, 95%CI: 3.13-50.22) as independent risk factors affecting treatment efficacy. |
| Limitations | 19 | Discuss limitations of the study, taking into account sources of potential bias or imprecision. Discuss both direction and magnitude of any potential bias | 8 | Several limitations exist in this study. First, as a single-center retrospective study, the generalizability and external validity of our findings require validation through multi-center data. Second, since the study population primarily consisted of Chinese neonates, caution should be exercised when extrapolating these results to other ethnic populations. Third, as an observational study, we could only demonstrate associations between rivaroxaban treatment and thrombus resolution, rather than establish causal relationships. Furthermore, although we adjusted for known confounding factors, there may be unidentified confounders influencing our results. Finally, the inability to conduct long-term follow-up for all patients limited our assessment of the treatment's long-term outcomes. |
| Interpretation | 20 | Give a cautious overall interpretation of results considering objectives, limitations, multiplicity of analyses, results from similar studies, and other relevant evidence | 8 | Retrospective data suggest that rivaroxaban is safe and effective in treating neonatal catheter-related thrombosis, with a higher complete thrombus resolution rate observed at 3 months compared to 6 weeks of anticoagulation therapy. Chemotherapy and difficult catheter placement were identified as independent risk factors affecting treatment efficacy. However, these findings require validation through multi-center, prospective, randomized controlled trials. |
| Generalisability | 21 | Discuss the generalisability (external validity) of the study results | 8 | Several limitations exist in this study. First, as a single-center retrospective study, the generalizability and external validity of our findings require validation through multi-center data. Second, since the study population primarily consisted of Chinese neonates, caution should be exercised when extrapolating these results to other ethnic populations. Third, as an observational study, we could only demonstrate associations between rivaroxaban treatment and thrombus resolution, rather than establish causal relationships. Furthermore, although we adjusted for known confounding factors, there may be unidentified confounders influencing our results. Finally, the inability to conduct long-term follow-up for all patients limited our assessment of the treatment's long-term outcomes. |
| Other information | |  | | |
| Funding | 22 | Give the source of funding and the role of the funders for the present study and, if applicable, for the original study on which the present article is based | 8 | This work was supported by the Startup Fund for scientific research, Fujian Medical University(Grant number：2022QH161). |

*Give information separately for cases and controls in case-control studies and, if applicable, for exposed and unexposed groups in cohort and cross-sectional studies.

**Note:** An Explanation and Elaboration article discusses each checklist item and gives methodological background and published examples of transparent reporting. The STROBE checklist is best used in conjunction with this article (freely available on the Web sites of PLoS Medicine at http://www.plosmedicine.org/, Annals of Internal Medicine at http://www.annals.org/, and Epidemiology at http://www.epidem.com/). Information on the STROBE Initiative is available at www.strobe-statement.org.
